# Fungal Beta-Glucosidases: A Bottleneck in Industrial Use of Lignocellulosic Materials

**DOI:** 10.3390/biom3030612

**Published:** 2013-09-03

**Authors:** Annette Sørensen, Mette Lübeck, Peter S. Lübeck, Birgitte K. Ahring

**Affiliations:** 1 Section for Sustainable Biotechnology, Aalborg University Copenhagen, A C Meyers Vaenge 15, 2450 Copenhagen SV, Denmark; E-Mails: aso@bio.aau.dk (A.S.); mel@bio.aau.dk (M.L.); psl@bio.aau.dk (P.S.L.); 2 Bioproducts, Sciences & Engineering Laboratory, Washington State University, 2710 Crimson Way, Richland, WA 99354, USA

**Keywords:** beta-glucosidase, cellulase, biomass, hydrolysis, biofuels

## Abstract

Profitable biomass conversion processes are highly dependent on the use of efficient enzymes for lignocellulose degradation. Among the cellulose degrading enzymes, beta-glucosidases are essential for efficient hydrolysis of cellulosic biomass as they relieve the inhibition of the cellobiohydrolases and endoglucanases by reducing cellobiose accumulation. In this review, we discuss the important role beta-glucosidases play in complex biomass hydrolysis and how they create a bottleneck in industrial use of lignocellulosic materials. An efficient beta-glucosidase facilitates hydrolysis at specified process conditions, and key points to consider in this respect are hydrolysis rate, inhibitors, and stability. Product inhibition impairing yields, thermal inactivation of enzymes, and the high cost of enzyme production are the main obstacles to commercial cellulose hydrolysis. Therefore, this sets the stage in the search for better alternatives to the currently available enzyme preparations either by improving known or screening for new beta-glucosidases.

## 1. Introduction

The ever-increasing energy consumption and the depletion of fossil resources have laid the foundation for a shift towards sustainable production of biofuels and bioproducts in biorefineries from renewable sources. Oil is currently the primary source of energy for the transportation sector and for production of chemicals and plastics. However, biorefineries are in the coming decades expected to supplement or replace oil refineries by maximizing biomass value, producing fuels and platform molecules for use as building blocks in the synthesis of chemicals and polymeric materials [1]. Biorefineries rely on the use of plant biomass in the form of dedicated energy crops or lignocellulosic agricultural residues as an abundant and inexpensive renewable energy resource [2]. Most biorefineries focus on production of a sugar platform of simple sugars that are released from biomass [1]. These sugars can then biologically or chemically be converted into fuels (e.g., ethanol, butanol and hydrocarbons), building block chemicals (e.g., different organic acids), as well as other high value bioproducts [3]. 

Plant biomasses are rich in lignocellulose which consists mainly of polysaccharides such as cellulose and hemicelluloses that together with the phenolic lignin polymer form a complex and rigid structure. The biomass composition depends on the plant/crop type, with cellulose being the most abundant component [4]. Cellulose is a long homogenous linear polymer of beta-D-glucosyl units linked by 1,4-beta-D-glucosidic bonds. The cellulose chains are assembled in larger rigid units held together by hydrogen bonds and weak van der Wall’s forces. Through parallel orientation, the chains form a highly ordered crystalline structure, but are interspersed with amorphous regions of more disordered structure [5,6,7].

The complex structure of the cellulose fibrils embedded in an amorphous matrix of lignin and hemicellulose strengthen the plant cell wall and give plants a natural recalcitrance to biological degradation. Pretreatment is crucial as a first step for increasing the accessibility of the biomass polymers for the following enzyme hydrolysis. The different pretreatment methods available are plentiful—including alkali-, acid-, or organic—solvent pretreatment, steam-, ammonia fiber- or CO_2_ explosion, and wet-oxidation [8,9,10]. The type of plant material as well as the severity of the pretreatment method applied will influence the characteristics of the lignocellulosic substrate for enzyme hydrolysis with regard to cellulose accessibility, degree of polymerization, hemicellulose content, lignin content, and other potential interfering compounds [11,12,13]. Such variation in biomass characteristics will influence the composition requirements for an optimal enzyme cocktail for the breakdown of different types of lignocellulosic biomasses [14]. Balanced enzyme cocktails and tailoring of enzymes for increased performance is of major importance for obtaining high yields of sugar monomers from hydrolysis, and especially the complete hydrolysis of cellulose is the main challenge that must be overcome. Among the cellulolytic enzyme complex, beta-glucosidases play a key role for the final conversion of cellobiose into glucose.

This review provides an overview of fungal beta-glucosidases in relation to industrial use of lignocellulosic materials. We discuss the significance of beta-glucosidases, how they represent the bottleneck in biomass conversion and the challenges in biomass hydrolysis in biorefineries.

## 2. Hydrolysis of Cellulose

The general biochemistry of cellulosic enzymatic hydrolysis has been reviewed extensively in previous literature [5,11,15,16] and will only be briefly discussed here.

The classical scheme for cellulose hydrolysis involves three main categories of enzymes: endo-1,4-beta-glucanases (EC 3.2.1.4), cellobiohydrolases (or exo-1,4-beta-glucanases) (EC 3.2.1.91), and beta-glucosidases (EC 3.2.1.21). Cellulose polymers are, through sequential and synergistic actions of these enzymes, degraded to glucose. The general consensus is that endo-glucanases randomly hydrolyze the internal 1,4-beta-linkages in primarily the amorphous regions of cellulose, rapidly decreasing the degree of polymerization. Cellobiohydrolases hydrolyze the cellulose polymer from the free ends, releasing cellobiose as product in a processive fashion, and finally, beta-glucosidases hydrolyze the cellobiose to glucose. Several fungal beta-glucosidases have furthermore been shown to produce glucose from larger cellodextrins, thus having the potential to increase the reaction rate and extent of cellulose hydrolysis [5,11,15,16].

The classical concept of cellulose hydrolysis described above has been agreed on for decades, but more recently, attention has been paid to accessory enzymes that are co-regulated or co-expressed by microbes during growth on cellulosic substrates. The crystalline chains in cellulose are tightly packed and additional factors are needed in order to make the substrate more accessible for the hydrolytic enzymes. Among such accessory enzymes are the GH61 proteins and the bacterial family 33 Carbohydrate Binding Modules (CBM33), which lack measurable hydrolytic activity, yet they are able to significantly enhance the activity of cellulases on pretreated biomass. Both proteins have flat substrate-binding surfaces and are capable of cleaving polysaccharide chains by oxidative reactions to disrupt the polymer packing, thereby increasing its accessibility [17,18,19,20].

The commercial viability of biorefineries has been burdened by the use of expensive enzymes needed to hydrolyze the biomass material after pretreatment [21,22]. It has been well established that producing higher concentration of sugars is an absolute necessity in an industrial setting as it lowers the heating requirements (lowering operating costs) and increases the volumetric efficiency (lowering capital costs) of the equipment [23]. Therefore, lowering the enzyme input and increasing the dry matter content during enzyme hydrolysis for higher cellulose conversion would be one of the most significant steps towards the direction of bioethanol production cost reduction and eventually leading to the commercialization of second generation biorefineries based on the lignocellulosic feedstock. 

Several researchers have worked on using corn stover for the bioethanol production. Karr *et al*. used lime pretreatment followed by enzymatic hydrolysis at 5% solids concentration (SC) and 20 FPU (Spezyme CP and Novozym 188) and obtained 60% cellulose conversion [24]. Kim *et al*. introduced ammonia recycle percolation pretreatment followed by enzyme hydrolysis at 1% SC and 10 FPU (Spezyme CP and β-glucosidase (Sigma-Aldrich, St Louis, MO, USA)) and obtained 92% cellulose conversion [25,26]. This concentration of solids will, however, be far from an industrial process. Bura *et al*. used SO_2_ catalyzed pretreatment followed by enzyme hydrolysis at 8% SC and 10 FPU (Spezyme CP, Novozym 188 and Multifect^®^ Xylanase) and obtained 100% cellulose conversion [27]. However, again the solid concentration was far lower than needed for operating any industrial process. Using chemicals such as sulfur could further affect the down-stream processing of products, and for instance, sulfur will be attached to the solid fraction remaining after sugar extraction [28]. Recently, Yang *et al*. used steam explosion pretreatment followed by enzyme hydrolysis at 25% SC and 20 FPU (Celluclast) and obtained 85% cellulose conversion [29]. Even though this study achieved high glucose concentrations, the amount of enzymes used was higher, affecting the applicability of the process. 

Cellobiohydrolases and endoglucanases are often inhibited by cellobiose [30], making beta-glucosidases important for avoiding product inhibition through conversion of cellobiose to glucose, and thereby, avoiding decreased hydrolysis rates of cellulose over time. However, beta-glucosidases are often themselves inhibited by their product glucose [31,32] making beta-glucosidase the rate-limiting enzyme. Maintaining a high hydrolysis rate of cellulose ultimately requires highly efficient beta-glucosidases that tolerate glucose at high levels. 

## 3. Enzymes: Past to Present

*Trichoderma reesei* is one of the most widely used species of filamentous fungi for the production of cellulolytic enzymes. The fungus was originally isolated during the Second World War where it was found to thrive on the US Army’s tent canvas (cellulose). Since the 1950s, the original strain of *T. reesei* has been subjected to multiple rounds of strain improvement for enhanced cellulase production, including increasing enzyme titers and reducing the catabolite repression effect as well as protease activity [33]. The fungal enzyme product, however, lacks sufficient beta-glucosidase activity for complete and efficient industrial cellulose hydrolysis [5,34]. 

Enhancement of the beta-glucosidase activity of the *T. reesei* enzyme product has been achieved through displacement of the native promoter by homologous recombination with xylanase and cellulase promoters obtaining a 4–7.5-fold increase in beta-glucosidase activity [35]. Other ways of increasing the beta-glucosidase activity of *T. reesei* include heterologous expression of beta-glucosidase from other fungi [36,37,38,39] thus creating a single expression host for the production of all relevant enzymes for converting cellulosic biomass into monomeric sugars.

Beta-glucosidases are widely produced by different genera and species of the fungal kingdom including Ascomycetes and Basidiomycetes, where especially the ascomycete genus *Aspergillus* has been widely studied for beta-glucosidase production. *A. niger* has been setting the standard in commercial beta-glucosidase production [40], but within the last few years more research papers have been published on efficient beta-glucosidases e.g., from other *Aspergillus* species and from the *Penicillium* genus [38,41,42]. 

Commercial enzyme preparations for cellulosic biomass hydrolysis were initially prepared as separate fungal fermentation products that needed to be combined for efficient hydrolysis, e.g., Celluclast (a *T. reesei* cellobiohydrolase and endo-glucanase product) and Novozym188 (an *A. niger* beta-glucosidase product) by Novozymes. More recently, the enzyme companies, Novozymes and Genencor, have replaced these two preparations with single products that contain the full array of enzymes for cellulosic biomass hydrolysis. Whether the products originate from strain improvement of the production strain to express all enzymes or if the products are mixes based on two or more fermentations is not disclosed by the companies. The optimal hydrolysis conditions of most commercial cellulosic enzymes are temperatures around 50 °C and a pH around 5. The enzyme loading must be optimized based on the biomass.

The current trend for the major enzyme companies is to team up with cellulosic biorefinery companies to specifically meet their needs in hydrolysis, working on optimizing the enzymes for a particular biomass and pretreatment method. Recently, Genencor has partnered with DuPont (http://biosciences.dupont.com), DSM with Poet (www.poetdsm.com), and Novozymes with Mossi & Ghisolfi Group (www.novozymes.com/en/news/news-archive/Pages/novozymes-partner-to-open-largest-cellulosic-ethanol-plant-in-2012.aspx), building the world’s first commercial-scale cellulosic ethanol plant in Crescentino, Italy. 

With enzymes being an expensive part of biomass processing, it would be of great interest to make enzyme production part of the processes within the biorefinery. Other research therefore looks at producing enzymes on-site to cut away the profit enzyme companies include in their pricing. By efficiently implementing enzyme production within the biorefinery, completing the value chain can be achieved by using streams within the biorefinery as fungal growth medium for enzyme production, and directly using this product (enzymes, fungus, and medium) in hydrolysis of biomass. This has already been shown for different fungi, e.g., *T. reesei* cultured on pretreated wheat straw [43], *A. niger* and *A. saccharolyticus* [44] cultured on the fiber waste fraction left after hydrolysis and fermentation, and *A. japonicus* cultured on castor bean meal waste for the biodiesel production [45]. 

Evaluating the overall production cost, the price of enzymes typically contributes to a large part of the total cost [37]. Efficient enzymes for lignocellulose degradation are, therefore, of high demand. As most of the currently used pretreatment methods remove lignin from the sugar polymers and in many cases also hydrolyze most of the hemicellulose, the main target for enzyme treatment is cellulose decomposition into glucose with beta-glucosidases being key enzymes in terms of complete cellulose hydrolysis.

## 4. The Bottleneck Enzyme: Beta-Glucosidase

Beta-glucosidases are most commonly classified based on either substrate specificity or nucleotide sequence identity. Beta-glucosidases hydrolyze the *O*-glycosyl linkage of terminal, non-reducing beta-D-glucosyl residues with release of beta-D-glucose, e.g., the bond in cellobiose. A wide specificity for beta-D-glucosides is found and there are examples of beta-glucosidases hydrolyzing beta-D-galactosides, alpha-L-arabinosides, beta-D-xylosides, or beta-D-fucosides [46]. Based on substrate specificity, beta-glucosidases have traditionally been divided into cellobiases (high specificity towards cellobiose), aryl- beta-glucosidases (high specificity towards substrates such as *p*-nitrophenyl-beta-D-glucopyranoside (pNPG)), or broad specificity beta-glucosidases [31,47]. Most beta-glucosidases are placed in the last category. 

A classification based on substrate specificity cannot sufficiently accommodate enzymes that act on several substrates; the best accommodation for this is the classification system proposed by Henrissat (1991) which is based on sequence and structural features [48]. The strength of this system especially lies in the investigation of the active site of the enzymes, with significant similarity of sequences being a strong indication of similarity in the fold of the structure, and analysis of the primary structure can thereby assign potential conserved active-site residues. Fungal beta-glucosidases are primarily placed in the family 3 glycosyl hydrolases with the active site signature pattern defined as written below, where the aspartate (D) is the active site residue involved in catalysis (underlined) [46,49]. 

GH3 active site signature:[LIVM](2) – [KR] – X – [EQKRD] – X(4) – G – [LIVMFTC] – [LIVT] – [LIVMF] – [ST] – D – X(2) – [SGADNIT]

Structural information is valuable for protein engineering purposes to improve enzyme activity and stability. Only a few GH3 beta-glucosidase structures have been solved and published: *Hordeum vulgare* (barley) [50], *Kluyveromyces marxianus* (a yeast) [51], *Thermotoga neapolitana* (a hyperthermophilic bacterium) [52], *Pseudoalteromonas sp*. (a marine bacterium) [53], a compost microbial community [54], and only recently, one crystal structure from a filamentous fungus: the *Aspergillus aculeatus* beta-glucosidase BGL1 [55]. Furthermore, the BGL1 of *T. reesei* is in the protein database PDB, but the accompanying research article has not been published. Homology modeling has been the method of choice for obtaining structural information from fungal beta-glucosidases which have no available crystal structures. The beta-glucosidases from *Aspergillus saccharolyticus* and *Penicillium purpugenum* were modeled prior to the availability of other fungal beta-glucosidase crystal structures, and even though the sequence identity was relatively low to the template structures used, it was obvious that the residues important for substrate binding and catalysis were conserved and that the distance between the catalytic residues is similar to that of other solved beta-glucosidases [38,56]. 

The solved structure of the fungal *A. aculeatus* BGL1 consists of three domains: a catalytic TIM (triosephosphateisomerase) barrel-like domain, an *α*/*β* sandwich domain, and a FnIII (fibronectin type III) domain. These domains are connected with two linker regions. The active site and the catalytic residues of AaBGL1 are located at the domain interface between the barrel and the *α*/*β* sandwich domains [55]. Hydrolysis of beta-1,4-glycosidic bonds by beta-glucosidases is carried out by an overall retaining double-displacement mechanism [57]. Two catalytic carboxylic acid residues at the active site facilitate the reaction with one carboxylic acid acting as a nucleophile and the other as an acid/base catalyst [58]. The catalytic nucleophile of GH3 family enzymes is always present at a specific structural location just after the *β*7 strand of the TIM barrel domain, however, the position and identities for the acid/base catalyst are not completely conserved but are rather phylogenetically variable, and thus, less readily divined [55,59]. The topology of the active sites of all glycoside hydrolases falls into three general classes: (i) pocket or crater, (ii) cleft or groove, and (iii) tunnel. Beta-glucosidases and non-processive exo-acting enzymes have a pocket or crater topology that is well suited for recognition of a saccharide non-reducing extremity [60], with the depth and shape of the pocket or crater reflecting the number of sub-sites that contribute to substrate binding and the length of the leaving group [61]. Hydrolytic activity towards cellodextrins is commonly reported for fungal beta-glucosidases [11], and compared to other beta-glucosidases, the structure of the *A. aculeatus* BGL1 active site has a long cleft extending from sub-site +1 which appears to be a more suitable binding pocket for cellooligosaccharides [55]. Meanwhile, the catalytic pocket of *A. saccharolyticus* BGL1 is wider than other beta-glucosidases as it is missing a loop structure by the active site. Amino acids at this loop have been described to have weak H-bonds with glucose at the -1 sub-site, thus the deletion of this loop may plan an important role in altering substrate accessibility as well as rapid release of the product from the enzymes [38]. 

Based on genomic data, fungal beta-glucosidases are often reported to have several putative glycosylation sites based on their predicted amino acid sequence. The crystal structure of the *A. aculeatus* BGL1 was found to be highly glycosylated by many large N-glycan chains, which is believed to facilitate increased resistance to proteolytic attack and contributes to protein stability [55].

## 5. Beta-Glucosidases in Biomass Hydrolysis: The Challenges

In relation to industrial biomass conversion, a good beta-glucosidase facilitates efficient hydrolysis at specified operating conditions. Key points to consider when evaluating a beta-glucosidase are hydrolysis rate, inhibitors, and stability, with product inhibition and thermal instability often being a restriction for maintaining high conversion rates throughout the hydrolysis. It is obvious that activity and stability varies among different beta-glucosidases. Previous papers have listed beta-glucosidases and their properties [47,62]; as an addition to this, in Table 1 we here present a list of some more recently characterized fungal beta-glucosidases.

**Table 1:** Beta-glucosidases and their properties.

Microorganism	Activity			Temp.	pH	Inhibition			Ref.
	Substrate	Km (mM)	Vmax (U/mg)	Opt. (°C)	Opt.	Substrate	Inhibitor	Ki (mM)	
*Aspergillus fumigatus* Z5	pNPG			60	6.0				[42]
*Aspergillus niger* NRRL 599	pNPG	3.11	20.83	60	4.8				[63]
*Aspergillus saccharolyticus*	pNPG	1.9	45	58	4.2				[38]
*Aspergillus terreus* NRRL 265	pNPG	2.5		60	5.0	pNPG	Glucose	13.6	[64]
	Cellobiose	3.7							
*Daldinia eschscholzii*	pNPG	1.52	3.21	50	5.0	pNPG	Glucose	0.79	[65]
*Fomitopsis palustris* FFPRI 0507	pNPG	0.117		70	4.5	pNPG	Glucose	0.35	[66]
	Cellobiose	4.81							
*Fusarium solani*	pNPG	1	55.6	65	4.5				[67]
*Humicola insolens*	pNPG	0.16	18.186.0		6.2				[68]
	Cellobiose	0.51							
*Neosartorya ficheri* NRRL181	pNPG	68	886	40	6.0				[69]
*Peniclillium funiculosum* NCL1	pNPG	0.057	1920	60	4.0–5.0	pNPG	Glucose	1.5	[70]
*Phoma* sp. KCTC11825BP	pNPG	0.3		60	4.5	pNPG	Glucose	1.7	[71]
	Cellobiose	3.2							
*Tolypocladium cylinndrosporum*	pNPG	0.85	85.23	60	2.4	pNPG	Glucose	39.5	[72]
*Trichoderma koningii* AS3.2774	pNPG	2.67		50	5.0				[73]
*Trichoderma reesei*	pNPG			70	5.0				[74]

Fungi naturally produce a broad array of lignocellulosic enzymes, and with more and more full genome sequences available, it becomes evident just how many different enzymes their genome encode for. However, the genetic code itself does not necessarily imply that the fungus is optimally expressing the needed enzymes for efficient biomass hydrolysis. For example, the amount and types of cellulases (GH5, 6, 7, 12, 45, 61) and associated hemicellulase activities (GH10, 11, 26, 29, 39, 62, 67, 74, 93) are relatively small in the genome of *T. reesei* compared with other ascomycetes, even though the fungus is one of the most efficient cellulose degraders known [75]. Function can be predicted from the genetic code, but profound expertise does not yet exist in linking gene sequence to the actual activity and efficiency of the encoded enzyme [76]. The pathway to this has been initiated through homology modeling based on known 3D enzyme structures. Structures of most enzyme families have been resolved, including beta-glucosidases as mentioned previously, and with more templates becoming available, homology modeling can predict the folds and activity of gene sequences. This knowledge is useful for enzyme optimization using protein engineering methods such as site-directed mutagenesis, e.g., for higher thermal stability [22]. However, most current research has focused on testing the performance of individual enzymes heterologously expressed and purified, free from contaminating activities, against each other, and studies on optimally balanced enzyme cocktails have been undertaken to identify the best combination and ratio of de novo enzyme mixtures for biomass hydrolysis [22,77]. However, among the difficulties in expressing the enzymes heterologously for studying their activities are that different hosts might alter the original glycosylation pattern in the enzyme, thereby seriously altering their activity and/or stability [78].

One area of focus that must be addressed is how to perform such enzyme screenings in high through-put systems on actual biomass samples [79]. In practical terms, when studying the activity and kinetics of beta-glucosidases, it is important to consider the substrate that is being used, as substrate specificity of beta-glucosidases varies [47,62,80,81,82] and the choice of substrate will influence the kinetic data obtained. Several different substrates with varying sensitivity and ease of use can be applied for the determination of beta-glucosidase activity. Some enzyme testing is currently done on artificial or purified substrates rather than complex biomasses. However, data obtained using synthetic biomass substrates or single purified components have little value and limited applicability in predicting and modeling real biomass hydrolysis [11]. Those substrates can be valuable in terms of studying specific activities, but conclusions should never be extended to actual biomass hydrolysis as it is often the case that activities are found to be lower due to reduced substrate accessibility as well as enzyme inhibitors.

High conversion rates are essential for efficient conversion of biomass. Accumulation of glucose during hydrolysis can significantly lower the rate of cellulose hydrolysis through inhibition by blocking the active site or preventing the hydrolyzed substrate from leaving [83]. In case of product inhibition (glucose), the effect is naturally increased during the course of the reaction as more and more glucose is formed, and for beta-glucosidases the end-product is generally not removed during hydrolysis so the actual reaction rate will differ more and more from maximum reaction rate. High tolerance of beta-glucosidases towards glucose accumulation is, therefore, of great importance. A broad range of data on inhibition by glucose is described in the literature, with several of the published K_i_ values collected in a table in the Handbook of Carbohydrate Engineering [47]. The K_i_ values reported range from below one to thousands. Even within the same fungal species, there is great variation in the extent of product inhibition reported for different beta-glucosidases [47]. Compounds other than glucose are potentially present in biomass that can be inhibitory and influence the activity of beta-glucosidases, including (but not exclusively) other simple sugars, sugar derivatives, amines, and phenols [84].

A decrease in the rate of glucose formation can also be caused by transglycosylation events as the enzyme reaction is a reversible process. Other than inhibiting the reaction by occupying the active site, glucose can also be considered to take part in transglycosylation, thus using the active site capacity in non-hydrolyzing action which will decrease the overall rate of hydrolysis. Transglycosylation is obviously an unwanted event in biomass hydrolysis, but it is frequently reported for beta-glucosidases [62]; especially at high substrate concentrations, the transglycosylation is observed [85]. Targeted mutagenesis aiming at displacing essential amino acids involved in transglycosylation could potentially reduce this mechanism [86]. 

Enzyme performance in actual biomass hydrolysis is affected by several factors including temperature, pH, and solids loading. First of all, the condition of the biomass is defined by the pretreatment method applied. Many pretreatment methods rely on high temperatures and acidic conditions to make the biomass accessible for enzyme hydrolysis. Enzymes will, depending on extremity and time of exposure be inactivated by pH and temperature variations. Ionic groups are involved in enzyme catalysis, such as the acid-base catalyst in the beta-glucosidase active site, and the protonation state of the carboxylic acid residue catalyst and the carboxylate nucleophile is essential for the enzymatic reaction, therefore, a pH change could impair the catalytic mechanism [87]. Beta-glucosidases perform well at pH 4–5 [47,62], but at pH much lower than that, a significant decrease in activity is found. Therefore, in most cases, the pretreated biomass must be pH adjusted to some degree as the acidity is usually beyond this. Regarding temperature, according to the van’t Hoff rule, reaction rates double with every 10 degrees Celsius increase of temperature, which applies to all chemical reactions including enzyme catalyzed reactions. However, when reaching high temperatures, protein stability will be affected, leading to denaturation, and thus irreversible inactivation of the enzyme. Mesophilic fungi that typically grow at 24–27 degrees Celsius are often times reported to produce beta-glucosidases with temperature optima around 60–75 degrees Celsius, and only moderate increases in thermal stability are seen in enzymes derived from thermophilic fungi [88]. Temperature and pH optimum for microbial beta-glucosidases have been reported in different reviews [47,62,88], but for biomass hydrolysis processes that typically run for the duration of several hours or even days, the stability of the enzyme at specified temperatures is important. Several papers claim to have discovered thermostable beta-glucosidases, however, often the activity was only verified at the high temperature for a short duration of time [88]. 

In industrial biofuel production, the pretreatment of biomass needs to be performed at very high dry matter content, above 20% (w/w), in order to increase product concentrations and decrease reactor volumes and distillation costs [23]. Most studies have, however, shown that hydrolysis rates decrease with increasing dry matter content in biomass hydrolysis. Suggested explanations for this are inefficient means of mixing, product inhibition, lignin or hemicellulose derivatives, or inhibition by adsorption of the enzymes to the biomass surface. Based on different correlation studies, it has been found that the adsorption effect best describes the decrease in hydrolysis with increase in solids’ loading [23]. It has further been recognized that enzyme performance is reduced by interaction with lignin or lignin–carbohydrate complex; however, of the cellulase and xylanase enzymes tested, beta-glucosidase was the least affected by lignin [89]. Attempts have been made to deal with this issue by adding non-enzyme proteins to the hydrolysis that will be absorbed by the biomass instead of the active enzymes [90]. Another more advanced solution would be to engineer the hydrophobicity of the surface amino acids on the enzymes to make them less prone to adsorption by the biomass.

Beta-glucosidases act on soluble substrates and are with regards to biomass hydrolysis highly dependent on the action of cellobiohydrolases and endoglucanases to provide substrate, as the beta-glucosidases cannot access the insoluble cellulose fibers. Meanwhile, cellobiohydrolases and endoglucanases are highly dependent on beta-glucosidases to maintain efficient hydrolysis by relieving product inhibition. Therefore, a balanced enzyme cocktail is essential for efficient hydrolysis of biomass. The optimal ratio of the enzymes will depend on the specific activity of the enzymes used, the condition of the biomass substrate (sugar accessibility) as well as physical reaction conditions [91]. The total amount of enzyme required directly reflects on cost. Economics of enzymatic hydrolysis has long been a topic of discussion and concern for the feasibility of lignocellulosic biomass conversion. Properties of new enzymes are continuously reported in literature as well as research on optimizing the enzyme cocktails for biomass hydrolysis with emphasis on using reduced enzyme loadings yet obtaining same hydrolysis efficiency. One strategy for resolving this is the minimal enzyme cocktail concept which concerns identification of the minimal number, the minimal levels, and the optimal combination of the best performing mono-active enzymatic activities to achieve degradation to monomeric sugar units [14]. Ideally, based on minimal enzyme cocktail concept studies, rather than using purified enzymes, a selected enzyme producing microorganism should be genetically modified with distinct promoters for each enzyme gene to facilitate optimal expression of each enzyme component. It should be ensured that the enzymes are correctly post-processed by such host microorganisms so that they are correctly folded and have optimal activity, stability, *etc*. Furthermore, such microorganisms should not have intra- or extracellular proteolytic activity that would affect enzyme expression. 

## 6. New and Improved Beta-Glucosidases

In order to optimize the use of different biomasses, it is important to identify new beta-glucosidases with improved abilities on the specific biomasses as well as with improved abilities such as stability and high conversion rates. As already discussed, product inhibition impairing yields, thermal inactivation of enzymes, and high cost of enzyme production are main obstacles of commercial cellulose hydrolysis and therefore set the stage in the search for better alternatives to the currently available enzyme preparations. The choice stands between screening for new beta-glucosidases and improving known beta-glucosidases. 

The number of fungal species on earth is estimated to 1.5 million of which as little as approximately 5% are known [92,93], a statement that calls for a more directed effort for unraveling the potential of unknown species found in nature. The identification and characterization of new fungal species are often encountered in literature. Within the black Aspergilli, to which several efficient beta-glucosidase products belong, several new species have been identified within recent years [94,95,96,97,98,99,100,101,102]. Screening for new enzymes can be performed at the genomic as well as the proteomic level—in either case, it can be a mixed gene or protein pool or a sample representing a specific species. The number of organisms being fully genome-sequenced is constantly increasing, and along with it, the sequences for new genes. Comparative searches in databases can reveal new beta-glucosidase sequences, but to know if they are better than current standard, they must be cloned, expressed, and assayed. Using a metagenomics approach, environmental DNA has been screened for beta-glucosidase activity with the findings of novel beta-glucosidases [103,104,105]. As another approach, screening secreted fungal proteins for new and improved beta-glucosidases has been reported with success, generally finding black Aspergilli to be superior [106,107].

Through genetic changes, enzymes can be tailored to obtain improved abilities. The changes can either be random by classical methods of mutagenesis or specifically targeted improvements aided by the solved crystal structures. 

The increased activity obtained from classical mutagenesis is most often due to changes at the regulatory level of enzyme expression leading to increased production of the gene of interest or decreased expression of conflicting genes and is therefore minded on production strain improvements, rather than changes to the enzyme itself for improved activity. One good example was the use of a combination of UV irradiation and nitrosomethyl guanidine treatment to develop the *T. reesei* strain RutC30 with improved total protein production and activity; one of the best existing *T. reesei* cellulase mutants [108].

Mutation, recombination, and selection set the stage for functional evolution in nature. Directed evolution mimics natural evolution by combining reiterative random mutagenesis and recombination with screening or selection for enzyme variants with improved properties [109,110,111]. Compared to classical mutagenesis, directed evolution targets a specific gene of choice with random changes being performed delimited to the gene of choice, followed by evaluation of the mutants [112]. Several publications exist on such strategy for non-fungal beta-glucosidases. For example, several single amino acid substitutions generated through error prone PCR were found to contribute to increased thermal resistance of *Paenibacillus polymyxa* beta-glucosidase that were then further recombined by gene shuffling [113]. The improvements of the final best clone were attributed to three mutations leading to formation of salt bridges and amino acids less prone to oxidation [114]. A similar approach of combining error prone PCR and gene shuffling was performed on *Pyrococcus furiosus* beta-glucosidase, generating an improvement of low temperature cellobiose hydrolysis [115]. More recently, gene shuffling of beta-glucosidases from *Thermobifida fusca* and *Paebibacillus polymxyxa* resulted in a mutant with increased thermostability compared to both parental enzymes, reported as a 144-fold increase in half-life of inactivation, and a 94% increase in k_cat_ towards cellobiose [116].

To perform more advanced mutagenesis, such as rational design, bioinformatics is a prerequisite. Protein structure can guide the fine-tuning of e.g., the active site by rational design by only a few specific mutations. A great amount of knowledge is available on the protein engineering possibilities for improving activity as well as stability [117]. With only a few filamentous fungal beta-glucosidase structures recently having been solved, most rational design has been performed on non-fungal beta-glucosidases. However, in a recent study, specific amino acids were mutated in the outer channel of the active site of a *T. reesei* beta-glucosidase to significantly improve activity as well as increase the thermostability [118]. 

## 7. Conclusions

Fungal beta-glucosidases are important enzymes in efficient hydrolysis of cellulosic biomass, as they relieve the inhibition of the cellobiohydrolases and endoglucanases by reducing cellobiose accumulation. They are key enzymes in the final part of biomass hydrolysis for producing the monomer sugars for the production of biofuels and platform molecules that can serve as building blocks in the synthesis of chemicals and polymeric materials. They are often the bottleneck in the process, and the most important challenge to overcome is product inhibition. To have a profitable biomass conversion process, the hydrolysis must yield high glucose concentrations and the beta-glucosidases must, therefore, not be inhibited by their product but maintain high conversion rates at high glucose concentrations. 
